# Reverse Engineering Targets for Recombinant Protein Production in *Corynebacterium glutamicum* Inspired by a Fast-Growing Evolved Descendant

**DOI:** 10.3389/fbioe.2020.588070

**Published:** 2020-12-09

**Authors:** Min Ju Lee, Jihoon Park, Kyunghoon Park, Jihyun F. Kim, Pil Kim

**Affiliations:** ^1^Department of Biotechnology, The Catholic University of Korea, Gyeonggi, South Korea; ^2^Department of Systems Biology, Division of Life Sciences, and Institute for Life Science and Biotechnology, Yonsei University, Seoul, South Korea

**Keywords:** *Corynebacterium glutamicum*, host for recombinant protein, iron consumption, cellular energy, glucose consumption, ribosome

## Abstract

We previously reported a *Corynebacterium glutamicum* JH41 strain with a 58% faster growth rate through application of adaptive laboratory evolution. To verify that the fast-reproducing strain was useful as a host for recombinant protein expression, we introduced a plasmid responsible for the secretory production of a recombinant protein. The JH41 strain harboring the plasmid indeed produced the secretory recombinant protein at a 2.7-fold greater rate than its ancestral strain. To provide the reverse engineering targets responsible for boosting recombinant protein production and cell reproduction, we compared the genome sequence of the JH41 strain with its ancestral strain. Among the 15 genomic variations, a point mutation was confirmed in the 14 bases upstream of NCgl1959 (encoding a presumed siderophore-binding protein). This mutation allowed derepression of NCgl1959, thereby increasing iron consumption and ATP generation. A point mutation in the structural gene *ramA* (A239G), a LuxR-type global transcription regulator involved in central metabolism, allowed an increase in glucose consumption. Therefore, mutations to increase the iron and carbon consumption were concluded as being responsible for the enhanced production of recombinant protein and cell reproduction in the evolved host.

## Introduction

Many attempts through microbial metabolic engineering have been made to overproduce metabolites by (1) improving the substrate uptake rate, (2) reducing the flux to undesired by-products, (3) introducing heterologous pathways and optimizing the activity of the enzyme, and (4) balancing product formation in cells, such as secretion of product to the extracellular medium (Yadav et al., 2012). However, the metabolic manipulation of microorganisms can cause flux imbalance due to the lack of understanding of the complex regulatory mechanisms essential for operation (Yadav et al., 2012). Adaptive laboratory evolution (ALE) is a reasonable approach because it not only copes with the physical defects of strains caused by genetic manipulation but also improves the productivity of cell factories (Sandberg et al., 2019; Lee and Kim, 2020). In addition, the commercialization of next-generation sequencing (NGS) technology and the development of omics tools have enabled reverse engineering by helping us better understand mechanisms of microbial regulation (Stella et al., 2019). Besides, genetically modified organism (GMO)-related issues in the food industry have increased the demand for non-GMO strains derived from ALE.

Growth rate is a significant parameter in evaluating the compatibility of industrial strains. It is generally recognized that fast growth rates can achieve high cell densities and high productivity (Feist et al., 2010). Genomic analyses of fast-growing mutant strains have revealed previously unknown metabolic engineering targets and methods to improve production hosts. Rugbjerg et al. (2018) reported that the introduction of the *rpoB* mutation into *Escherichia coli* increased the growth rate by 13% while also increasing the production rate of mevalonate, a building block of terpenoid products such as fragrances and bioplastics, by 71%. In addition, Wang et al. reported that the introduction of *gntR1* and *ramA* mutations into *Corynebacterium glutamicum* increased the growth rate by 37% and the productivity of lysine by 100% (Wang et al., 2018).

Recently, we reported a fast-reproducing *C. glutamicum* JH41 strain, obtained through the application of ALE (Park et al., 2020). The transcriptome pattern of the evolved JH41 strain revealed that the upregulation of genes in the tricarboxylic acid cycle (TCA) cycle and respiratory chain, and oxidative stress responses allowed the growth promotion. Since *C. glutamicum* is a useful host for recombinant protein expression (Lee and Kim, 2018), it was proposed that the fast-reproducing JH41 strain might be an improved host for recombinant protein production and may also provide hidden genetic targets for reverse engineering of *C. glutamicum* hosts. In this report, the fast-reproducing JH41 was indeed verified as an improved host for recombinant protein production. Genomic analysis of the evolved strain was therefore performed to explain the reasons for the increased growth rate and recombinant protein production.

## Materials and Methods

### Strains and Plasmids

*Corynebacterium glutamicum* PT strain (ancestor of JH41) and a fast-reproducing JH41 strain (descendant of PT) were used as hosts for recombinant protein production and genomic analysis (Park et al., 2020). DNA manipulation followed the method described by Sambrook et al. using *E. coli* DH10B (Invitrogen Inc., Carlsbad, CA, United States) (Sambrook and Russell, 2001). The strains and plasmids are listed in Table 1 and the primers in Supplementary Table 1. Target gene-disrupted or gene-substituted *C. glutamicum* strains were prepared by double crossover using the pK*mobsacB*-based plasmids. Homologous arms of the target genes were constructed by connecting two fragments amplified from genomic DNA using overlap extension PCR (Ho et al., 1989). Oligonucleotide synthesis and plasmid sequence confirmation were performed at a facility of Bionics Inc., (Seoul, South Korea). *pfu* polymerase was purchased from Solgent Inc., (Daejeon, South Korea) and restriction enzymes and T4-ligase from New England Biolabs Inc., (Ipswich, MA, United States).

**TABLE 1:** Strains and plasmids used in this study.

Strains and plasmids	Description	References
**Strains *E. coli***		
DH10B	F^–^ *mcrA* Δ(*mrr-hsd*RMS-*mcrBC*) Φ80*lacZ*ΔM15 Δ*lacX74 recA1 endA1 araD139*Δ(*ara leu*)7697 *galU galK rpsL nupG* λ^–^	Invitrogen
BL21(DE3)	*E. coli* F^–^*ompT gal dcm lon hsdS*_B_ r_B_^–^ m_B_^–^; *E. coli* B strain), with λDE3	Novagen
*C. glutamicum* PT	Home stock of *C. glutamicum* wild-type, Biotin auxotroph, derived from KCTC No. 1445	Kim et al., 2016
JH41	A descendant of *C. glutamicum* PT, selected after 600 generation, 58% faster reproduction than PT	Park et al., 2020
PT NCgl0774:35-bp	PT harboring genomic NCgl0774, putative siderophore- binding lipoprotein A, inserting the 35-bp sequence	This study
PT NCgl1159^C–108T^	PT harboring genomic NCgl1159, F_0_F_1_-ATP synthase subunit A, substituting the 108th upstream sequence of NCgl1159 from C to T	This study
PT Δ*ripA*	PT derivative, Δ*ripA* (0.8-kb deletion)	This study
PT Δ*dtxR*	PT derivative, Δ*dtxR* (0.9-kb deletion)	This study
PT *ramA*^A239G^	PT harboring genomic *ramA*, transcriptional regulator MalT, substituting to A239G	This study
PT SBP B^C–14G^	PT harboring genomic SBP B (NCgl1959), putative siderophore-binding lipoprotein, substituting the 14^h^ upstream sequence of SBP from C to G	This study
PT S + R	PT harboring genomic mutation of PT *ramA*^A239G^ and PT SBP B^C–14G^	This study
**Plasmids**		
pET24a	*E. coli* expression vector, P_T__7_, Km^R^	Novagen
pET24a-DtxR	pET24a harboring DtxR (NCgl1845) of *C. glutamicum*	This study
pSL360	*E. coli* and *C. glutamicum* shuttle vector, Km^R^	Park et al., 2004
pCG-H36A	*C. glutamicum* vector, TAT-signal sequence of cg1514 (start codon is changed to ATG), a strong synthetic H36 promoter	Yim et al., 2016
pCG-H36A-agarase	pCG-H36A derivative, harboring *S. coelicolor* agarase	Yim et al., 2016
pCG-H36A(-SS)	pCG-H36A derivative, signal peptide deleted vector, H36 promoter	This study
pCG-H36A(-SS)-GFP	pCG-H36A(-SS) derivative, harboring eGFP	This study
pCG-H36A-*porD*	pCG-H36A derivative, TAT-signal sequence of *porD*, H36 promoter	This study
pCG-H36A-*porD-*GFP	pCG-H36A-*porD* derivative, harboring eGFP	This study
pK19*mobsacB*	Suicide vector for double recombination, Km^R^, *sacB* of *Bacillus subtilis*	Schafer et al., 1994
pSL360-SBP B	pSL360 harboring putative siderophore-binding lipoprotein, SBP B (NCgl1959) of *C. glutamicum*	This study
pK19*mobsacB* -NCgl0774:35-bp	pK19*mobsacB* harboring homologous arm for insertion of NCgl0774:35-bp	This study
pK19*mobsacB* -Δ*ripA*	pK19*mobsacB* harboring homologous arm for deletion of Δ*ripA*	This study
pK19*mobsacB* -Δ*dtxR*	pK19*mobsacB* harboring homologous arm for deletion of Δ*dtxR*	This study

### Media and Batch Cultures

As for the preculture for DNA manipulations, lysogeny broth (LB) complex medium was used for *E. coli*, and brain heart infusion (BHI) medium was used for *C. glutamicum*. *C. glutamicum* was incubated at 30°C and 200 rpm in a shaking incubator, and *E. coli* was incubated at 37°C and 220 rpm. For batch cultures of *C. glutamicum* strains, a modified MCGC minimal medium [0.9% glucose, 6 g Na_2_HPO_4_, 4 g (NH4)_2_SO_4_, 3 g KH_2_PO_4_, 1 g sodium citrate dehydrate, 1 g NaCl, 0.1 g MgSO_4_–7H_2_O, 20 mg FeSO_4_–7H_2_O, 2 mg MnSO_4_–H_2_O, 2 mg FeCl_3_, 1 mg thiamine–HCl, 200 μg biotin, 35 μg CaCl_2_, 0.5 μg ZnSO_4_–7H_2_O, 0.2 μg Na_2_B_4_O_7_–10H_2_O, 0.2 μg CuCl_2_–2H_2_O, 0.1 μg (NH_4_)_6_Mo_7_O_24_–4H_2_O per liter] was used (Osten et al., 1989). A 500-ml baffled flask containing 50 ml of the modified MCGC medium was used for efficient oxygen transfer. The initial cell mass was adjusted to OD_600_ = 0.1. Biomass was estimated by measuring optical density (OD) at 600 nm every hour and converting into g_–__DCW_/L unit by the coefficient of 0.25.

### Secretory Production of Recombinant Protein

To confirm the recombinant protein production of the hosts, pCG-H36A-agarase encoding a strong autonomous promoter and TAT-secretion signal sequence with the agarase gene from *Streptomyces coelicolor* was further transformed into the PT and JH41 strains (Yim et al., 2016). After incubating for 24 and 48 h in a 500-ml baffled flask containing 50 ml of MCGC media, 50 ml of the culture broth was centrifuged (12,000 × g, 4°C, 5 min). The supernatant containing the secreted protein was concentrated 50-fold using a centrifugal filter (Amicon^®^ Ultra-15 10K, Merck Millipore, Burlington, MA, United States), and then, the buffer was changed with 50 mM Tris–HCl (pH 7.0). The prepared protein samples were diluted to final OD_600_ values and then loaded on a 12% sodium dodecyl sulfate–polyacrylamide gel electrophoresis (SDS-PAGE) gel. The photograph of the gel was captured using a chemical fluorescence image analyzer (Chemi Doc MP System, Bio-Rad Laboratories, Inc., Hercules, CA, United States), and the intensity of the target band was compared using the ImageJ program.

### Genome Sequencing

For genome sequencing, genomic DNAs of *C. glutamicum* PT and JH41 were isolated using a gDNA prep kit (Solgent Inc., Daejeon, South Korea) according to the manufacturer’s instructions. The genome sequence was determined using the PacBio RS II (Pacific Biosciences Inc., Menlo Park, CA, United States) at a DNA sequencing facility (ChunLab Inc.). Raw sequences were assembled with PacBio SMRT Analysis ver. 2.0 software (Pacific Biosciences Inc., Menlo Park, CA, United States). Gene prediction was performed using Glimmer 3 (Delcher et al., 2007), and annotations were performed by a homology search against the SEED database, Universal Protein Resource (UniProt) database, and eggNOG database (Disz et al., 2010). Genome sequences of the PT and JH41 strains were compared using EzGenome (http://ezgenome.ezbiocloud.net/ezg_browse) and CLgenomics program (ChunLab). Analysis of single-nucleotide polymorphisms (SNPs) and gap was performed using the MAUVE (Darling et al., 2004) and MegaX (Kumar et al., 2018) programs.

### Analyses of ATP, Iron, and Glucose Concentrations

Cells were harvested from batch cultures (1 ml) at 5 h by centrifugation (8,000 × *g*, 4°C, 5 min) and washed twice with sterile distilled water. The cell suspension (0.8 ml) was mixed with 0.2 g of glass beads (212–300 μm) in a screw-capped tube followed by homogenization for 30 s using a bead beater (Model 607, BioSpec Inc., Bartlesville, OK, United States). After chilling in an ice bath for 1 min, homogenization was repeated five more times. Cell debris was removed by centrifugation (8,000 × *g*, 4°C, 5 min), and the supernatant was used for measuring intracellular ATP.

ATP concentration was estimated using an ENLITEN ATP Assay System Bioluminescence Detection Kit (#FF2000, Promega Inc., Madison, WI, United States). Light intensity was detected by a luminometer (GloMax^®^-96 Microplate Luminometer, Promega Inc., Madison, WI, United States) after a luciferase-driven reaction (Na et al., 2015).

Iron consumption was estimated by measuring the remaining free iron concentration using the *o*-phenanthroline colorimetric method (Serra et al., 2015) after cell removal. Culture broth (1 ml) was centrifuged (12,000 × *g*, 4°C, 5 min), and the supernatant (100 μl) was mixed with 20 μl of bromophenol blue (0.4 g/L). After pH adjustment to 3.5 by adding 0.2% H_2_SO_4_, a further 10 μl of hydroquinone (1 g/L), 20 μl of *o*-phenanthroline (2.5 g/L), and distilled water (up to 250 μl) were added to the mixture. After 1 h of color development at room temperature, absorbance was measured at 510 nm. The analyses of ATP and iron concentrations were determined at least three times with three biological replicates.

To analyze the glucose consumption of JH41 and PT *ramA*^A239G^, the glucose concentration in the medium was measured by a reducing sugar quantification method using a 3,5-dinitrosalicylic acid (DNS) reagent (Deshavath et al., 2020). After cell removal, 150 μl of supernatant and 150 μl of DNS reagent were mixed and heated at 100°C for 5 min. After standing to cool for 10 min at room temperature, 200 μl of the mixture was dispensed into 96-well plates to measure absorbance at 540 nm. D-Glucose reagent was used as a standard.

### Determination of DtxR Affinity on the Mutated Promoter Sequence

The affinity of negative regulator diphtheria toxin repressor (DtxR) on the mutated promoter region was determined by an electrophoresis mobility shift assay using the purified DtxR protein and the PCR-amplified DNA fragment. The *dtxR* gene from *C. glutamicum* was cloned and expressed in *E. coli* BL21 (DE3) with an additional 12 × His tag in the expression plasmid (pET24b-*dtxR*, Table 1). The protein was further purified using Ni^2+^ chelate affinity chromatography, as described previously (Wennerhold et al., 2005). To test the binding of DtxR to the putative target promoter DNA, the purified DtxR protein (0–350 ng, dimeric form) was dissolved with PCR-amplified DNA fragments (399 bp, 200 ng) in a 10-μl binding buffer [50 mM Tris–HCl (pH 7.5), 5 mM MgCl_2_, 40 mM KCl, 5% (v/v) glycerol, 1 mM dithiothreitol (DTT), and 150 μM MnCl_2_]. The reaction mixture was loaded onto a 15% native polyacrylamide gel containing 1 mM DTT and 150 μM MnCl_2_ after incubation for 30 min at room temperature. Electrophoresis was performed at room temperature and 170 V using 1 × TB (89 mM Tris base, 89 mM boric acid) supplemented with 1 mM DTT and 150 μM MnCl_2_ as an electrophoresis buffer. The gel was then stained with a fluorescent dye (SYBR Green I EMSA Kit, Invitrogen Inc., Carlsbad, CA, United States) according to the manufacturer’s instructions. The photograph of the gel was captured using a chemical fluorescence image analyzer (Chemi Doc MP System, Bio-Rad Laboratories, Inc., Hercules, CA, United States).

### Transcription Quantification of rRNAs

The ribosomal RNAs (rRNAs) transcription level determination method was followed as described (Kwon et al., 2015). Total RNA was extracted from actively growing *C. glutamicum* strains using TRIzol1 reagent (Invitrogen, Carlsbad, CA, United States) and NucleoSpin1 RNA II Kit (Macherey-Nagel, Düren, Germany) after cell disruption using a Mini-Beadbeater-16 (BioSpec, Bartlesville, PA, United States) with glass beads (acid washed, 212–300 mm, Sigma-Aldrich, MO, United States). The 50 ng of total RNAs was used for complementary DNA (cDNA) synthesis using ReverTra Ace-α-1 (Toyobo, Osaka, Japan) according to the manufacturer’s instructions. THUNDERBIRD SYBR1 qPCR Mix (Toyobo, Osaka, Japan) and the Step-One Plus Real-Time PCR System (Applied Biosystems, Foster City, CA, United States) were used for the gene expression analysis. Step-One Plus Software ver. 2.0 (Applied Biosystems, Foster City, CA, United States) was used to quantify the relative transcription. *gapA* [glyceraldehyde 3-phosphate dehydrogenase (GAPDH)] and *leuA* (2-isopropyl malate synthase) genes were used as internal references. The relative expression of rRNAs with the reference genes was determined using the 2^–Δ^ ^Δ^ ^CT^ (Livak) method. Primers for identifying transcription levels are shown in Supplementary Table 1.

## Results

### Effect of Fast-Growing JH41 Host on the Secretory Production of Recombinant Protein

**FIGURE 1:**
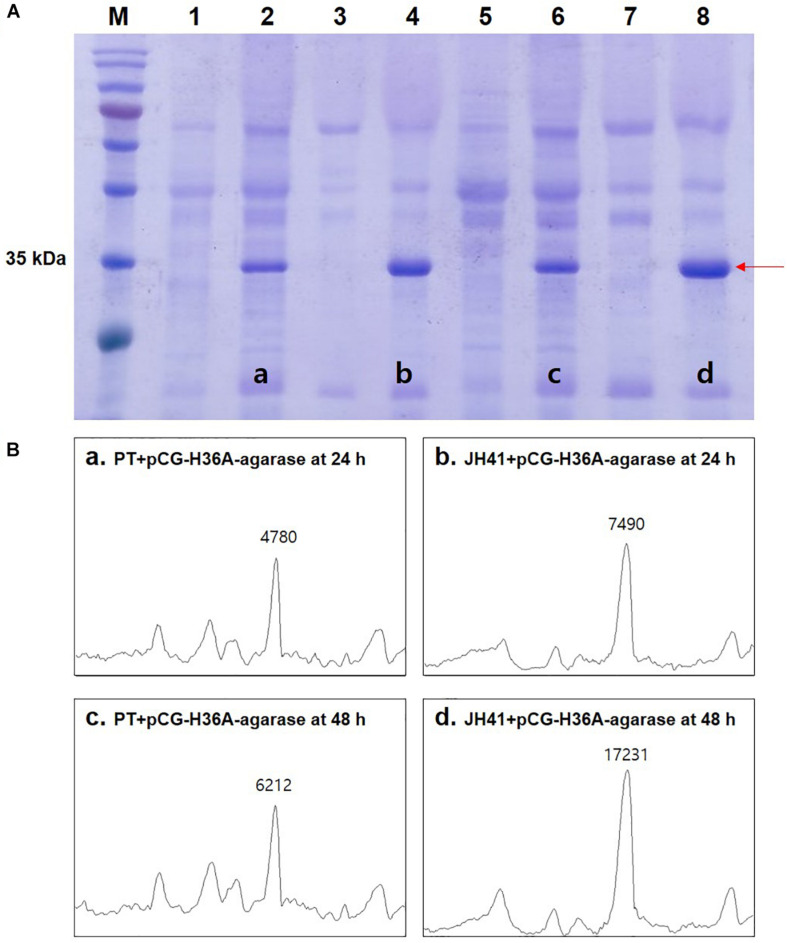
Effects of host on the secretory production of recombinant protein. **(A)** Image of sodium dodecyl sulfate–polyacrylamide gel electrophoresis (SDS-PAGE). Lane 1, PT + pCG-H36A (empty vector) at 24 h; lane 2, PT + pCG-H36A-agarase at 24 h; lane 3, JH41 + pCG-H36A (empty vector) at 24 h; lane 4, JH41 + pCG-H36A-agarase at 24 h; lane 5, PT + pCG-H36A (empty vector) at 48 h; lane 6, PT + pCG-H36A-agarase at 48 h; lane 7, JH41 + pCG-H36A (empty vector) at 48 h; lane 8, JH41 + pCG-H36A-agarase at 48 h. Arrow indicates the secreted agarase band (34 kDa). The image is from the representative experiment from three independent experiments. **(B)** Band density estimation of the SDS-PAGE image using ImageJ software. a: lane 2; b: lane 4; c: lane 6; d: lane 8.

Because protein is the major macromolecule in cells, the rapidly reproducing JH41 strain was supposed to be able to produce a protein at an enhanced rate. The fact that no extracellular protease activity or economical purification of secretory protein was found in *C. glutamicum* host (Lee and Kim, 2018) further encouraged us to hypothesize that the rapidly reproducing JH41 strain would become an improved *C. glutamicum* host for secretory protein production. To verify this hypothesis, a plasmid designed to secrete foreign agarase (pCG-H36A-agarase) was introduced into JH41 and PT strains. The secretory recombinant protein (agarase, 34 kDa) showed a major band in the SDS-PAGE (Figure 1A). The band intensities of the recombinant protein from the JH41 host were 7,490 AU at 24 h and 17,231 AU at 48 h, while those from the PT host were 4,780 AU at 24 h and 6,212 AU at 48 h (Figure 1B). The JH41 host produced the secretory recombinant protein (56% at 24 h and 177% at 48 h) at a greater rate than the PT host. In order to confirm the production effect of other proteins in the JH41 host, a plasmid expressing green fluorescent protein (GFP) in cytoplasm (pCG-H36A[-SS]-GFP, no signal sequence) and a plasmid secreting GFP by a TAT-signal sequence (pCG-H36A-*porD*-GFP) were introduced into the JH41 host (Supplementary Figure 1). The productions of cytoplasmic GFP and secreted GFP from the JH41 host were greater than those from the PT host by 5–16% and 13–116%, respectively. Therefore, the rapid-reproducing JH41 strain was an enhanced host for the production of recombinant proteins.

### Genome Analysis of the JH41 Host

To understand the reasons for enhanced cell reproduction and to provide reverse engineering targets responsible for the improved recombinant protein production, the JH41 genome was resequenced and deposited [National Center for Biotechnology Information (NCBI) accession number PRJNA554987]. A total 15 genomic mutations were found to be accumulated in JH41 compared to its ancestral PT (Table 2). Among them, four were found in the non-coding region, including an internal transcribed spacer (ITS) mutation, and 11 in the coding region. Genomic mutations were categorized into the clusters of orthologous groups of proteins (COG) as four to the function unknown group (S), two to the transcription group (K), two to the inorganic ion transport and metabolism group (P), one to the defense mechanism (V), one to the signal transduction mechanism (T), and one to the energy production and conversion group (C). The remaining three mutations did not show protein function or were not protein-encoding regions. A 35-bp repeated mutation in the structural gene encoding a putative siderophore-binding lipoprotein A (SBP A, NCgl0774) and a substitution mutation (C→G) at the 14 bases upstream of the start codon of the gene encoding a putative siderophore-binding lipoprotein B (SBP B, NCgl1959) were identified. A condensed mutation was also found in one of the operons encoding rRNA primary transcript. One mutation was found in the 16S rRNA structural gene, and another 23-point mutations were densely observed between the 16S and 23S rRNA sequences, which are called 16S–23S internal transcribed spacer (ITS) (Osorio et al., 2005).

**TABLE 2:** Genomic mutations of the JH41 host over ancestral host.

PT position^a^	JH41 position^a^	COG functional category^b^	Locus tag	Mutation region	Variants	Function (gene name)	mRNA fold based on transcriptome^c)^
80,225	1,076,088	S	NCgl2726	Non-coding (-57 bp of NCgl2726)	Substitution (G→A)	Tripartite tricarboxylate transporter substrate binding protein	3.05
461,943	1,457,809	-	-	16S rRNA coding region	Substitution (A→G)	Bacterial small subunit ribosomal RNA; 16S ribosomal RNA	–
462,350	1,458,217			Non-coding (16S-23S ITS of 1^st^ rRNA operon)	Similar to 16S–23S ITS of 5th rRNA operon	rRNA operon 16S-23S ITS (internal transcribed spacer)	–
1,191,984	2,187,859	T	NCgl0722	Coding (372 bp)	Insertion (C)	Histidine protein kinase (*mtrB*)	1.27
1,251,401	2,247,312	P	NCgl0774	Coding (15 bp)	Repeat (35 bp, AAAACTCTCCTCCGCA CTGATCGTGCTTCTCG CAG)	ABC transporter system; A putative Fe^3+^-siderophore-binding lipoprotein	0.03
1,660,001	2,655,914	C	NCgl1159	Non-coding (-108 bp of NCgl1159)	Substitution (C→T)	ATP synthase F_0_F_1_ subunit A	0.27
1,968,380	2,964,295	K	NCgl1435	Coding (201 bp)	Substitution (A→G)	Transcriptional regulator	1.17
2,004,381	3,000,298	–	–	Coding (67 bp)	Substitution (G→A)	Hypothetical protein	–
2,326,488	217,999	P	NCgl1959	Non-coding (-14 bp of NCgl1959)	Substitution (C→G)	ABC transporter system; a putative Fe^3+^-siderophore-binding lipoprotein	5.1
2,521,778	413,291	S	NCgl2129	Coding (110 bp)	Substitution (A→T)	Uncharacterized protein, cell membrane; membrane; signal; transmembrane; transmembrane helix.	1.43
2,589,950	481,463	S	NCgl0891	Coding (709 bp)	Substitution (T→G)	HNH endonucleases	1.08
2,589,952	481,465	S	NCgl0891	Coding (711 bp)	Substitution (G→T)	HNH endonucleases	1.08
2,708,045	599,558	K	NCgl2298	Coding (757 bp)	Substitution (G→A)	HTH-type transcriptional repressor (*osrR*)	1.27
2,743,443	634,956	V	NCgl2331	Coding (610 bp)	Substitution (G→A)	Penicillin binding protein	0.53
2,899,770	791,283	K	NCgl2472	Coding (239 bp)	Substitution (T→C)	Transcriptional regulator MalT (*ramA*)	1.17

*^a^Genome sequence data NCBI bioproject number: PRJNA554987. ^b^COG functional categories (M, cell wall/membrane/envelope biogenesis; T, signal transduction mechanisms; V, defense mechanisms; K, transcription; L, replication, recombination and repair; C, energy production and conversion; E, amino acid transport and metabolism; P, inorganic ion transport and metabolism; S, function unknown). ^c^Data adopted from the transcriptome (NCBI bioproject number: PRJNA556334) (Park et al., 2020).*

### Mutation Effect on Iron Consumption

**FIGURE 2:**
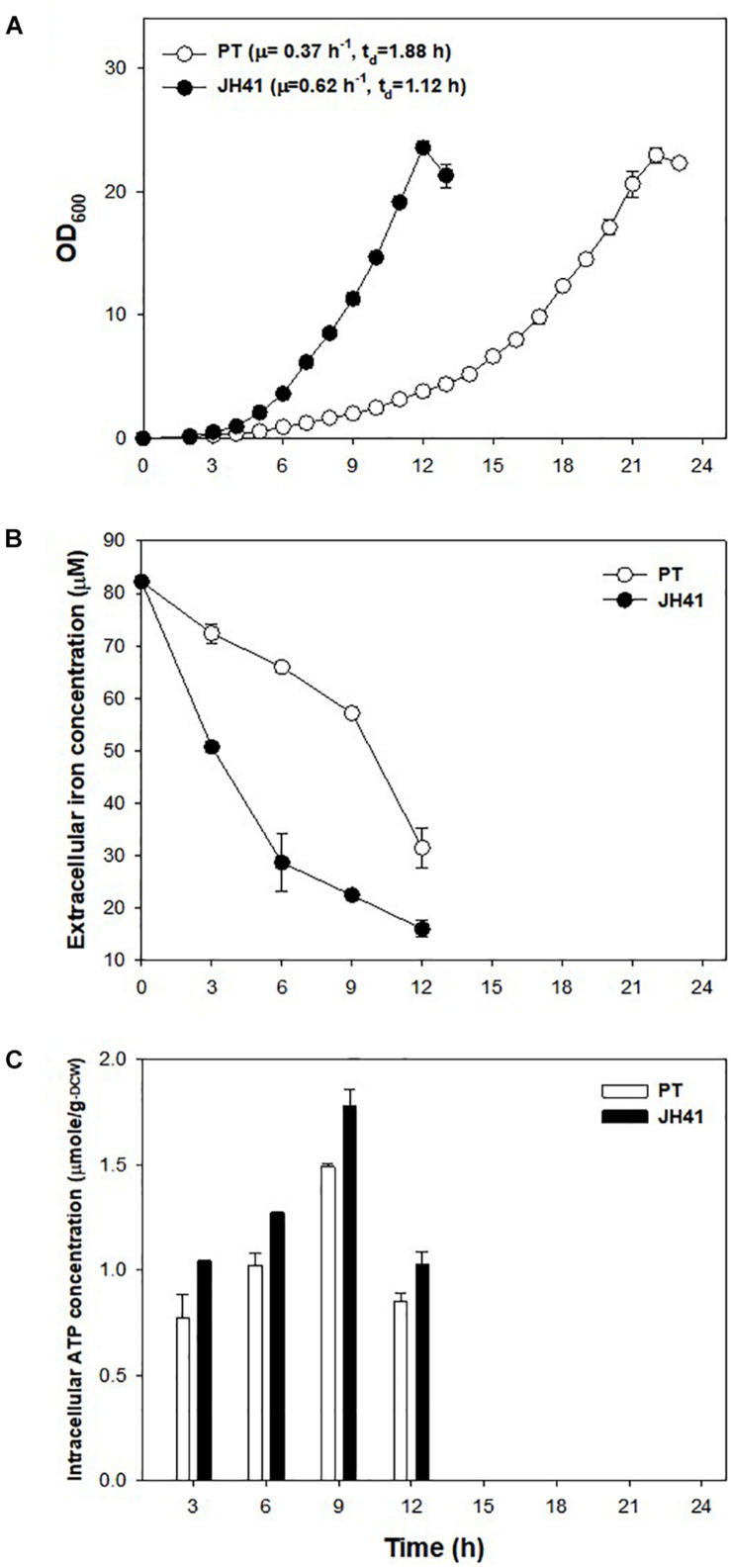
Profiles of growth, iron consumption, and intracellular ATP concentration of the JH41 host. **(A)** Batch culture growth profiles of PT and JH41 hosts. Cultures were in a 500-ml baffled flask containing 50 ml of modified MCGC medium. Each data point represents the mean ± SD; *n* = 3 biologically independent samples. (PT: white; JH41: black). **(B)** Iron consumption in PT and JH41 hosts. Samples were taken from the batch cultures (500-ml baffled flask containing 50 ml of modified MCGC medium). Iron concentration of the medium was measured by the *o*-phenanthroline colorimetric method. Error bars show a statistical mean ± SD; *n* = 3 biologically independent samples. (PT: white; JH41: black). **(C)** Intracellular ATP concentrations in PT and JH41 strains. Samples were taken from the batch cultures (500-ml baffled flask containing 50 ml of modified MCGC medium). Intracellular ATP was measured by the luciferin–luciferase reaction immediately after disrupting cells. Error bars show a statistical mean ± SD; *n* = 3 biologically independent samples (PT: white; JH41: black).

The mutations observed in two presumed siderophore-binding lipoproteins (NCgl0774: 35-bp repeated mutation; NCgl1959: point substitution mutation at -14 bp) suggested their possible role in the rapid reproduction of JH41. To explore the functional significance of these putative iron-importing systems (Ribeiro and Simões, 2019), the iron consumption of JH41 was profiled (Figure 2B). Iron concentration in the medium decreased more rapidly in the JH41 strain. Since iron is an essential element for bacteria and acts as a redox center for several cytochromes and iron–sulfur proteins in the electron transfer chain (Hider and Kong, 2010), it was assumed that the rapidly reproducing JH41 might harbor greater energy than its ancestor. To validate this, ATP concentrations of the fast-growing JH41 and ancestral PT were analyzed. Indeed, the intracellular ATP concentrations of JH41 were greater than those of the PT strain with a range of 19–35% during the active growing phase (Figure 2C).

To assess which of the putative siderophore-binding lipoproteins affected iron consumption, a PT strain harboring the genomic NCgl0774 gene with the precise 35-bp repeated sequence (PT NCgl0774:35-bp) was constructed for complementation tests. The constructed PT NCgl0774:35-bp strain showed no differences compared with the PT strain in the growth profile (Supplementary Figure 2). This result indicated that the 35-bp repeat mutation on the putative siderophore-binding protein A was not a prominent factor contributing to the rapid reproduction of JH41.

**FIGURE 3:**
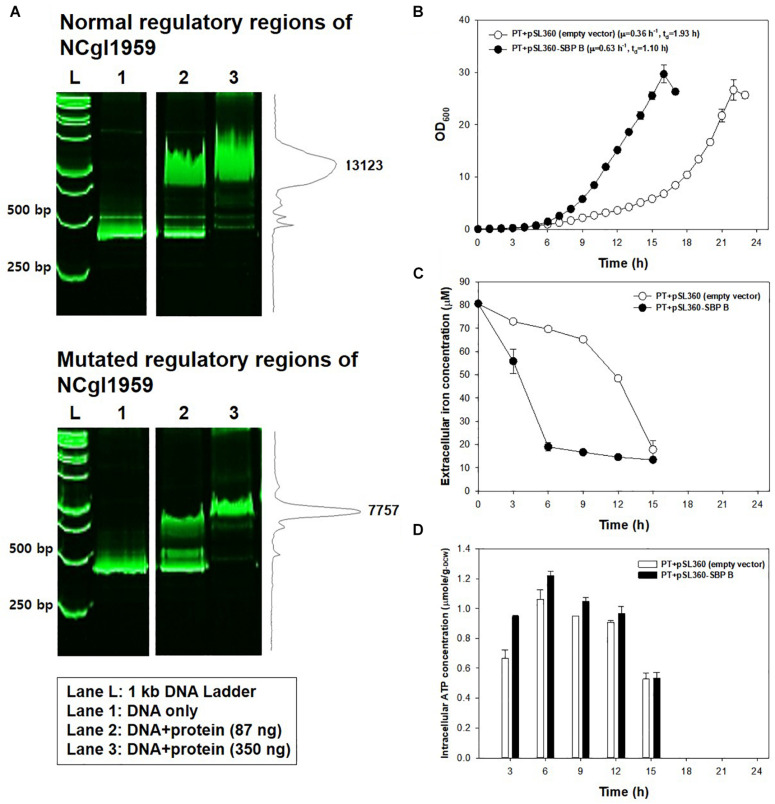
Effects of the mutation at 14 bases upstream of NCgl1959. **(A)** Native polyacrylamide gel image showing the affinity of DtxR protein to the normal DNA fragment (up) and to the mutated DNA fragment (down). L: size marker; lane 1: DNA only; lane 2: DNA + DtxR (87 ng); lane 3: DNA + DtxR (350 ng). The DNA fragments were stained with SYBR Green. The chromatogram on the right side represents the intensity of bands by ImageJ software. The image is from the representative experiment from three independent experiments. **(B)** Growth profile of PT strain harboring plasmid constitutively expressing NCgl1959. Each data point is the mean ± SD; *n* = 3 biologically independent samples. (PT + empty vector: white; PT + pSL360-SBP B: black). **(C)** Iron consumption in PT strain harboring plasmid constitutively expressing NCgl1959. The iron concentration in the medium was measured by the *o*-phenanthroline colorimetric method. All strains were cultured in a 500-ml baffled flask containing 50 ml of modified MCGC medium. Bar heights and error bars show a statistical mean ± SD; *n* = 3 biologically independent samples (PT + empty vector: white; PT + pSL360-SBP B: black). **(D)** Intracellular ATP concentrations in PT strain harboring plasmid constitutively expressing NCgl1959. The intracellular ATP luciferin–luciferase reaction was measured immediately after harvest during the actively growing phase. Point and error bars show a statistical mean ± SD; *n* = 3 biologically independent samples (PT + empty vector: white; PT + pSL360-SBP B: black).

Another point mutation found in the JH41 strain was located at -14 bp of the start codon of NCgl1959 encoding a putative siderophore-binding lipoprotein B, in the middle of the consensus sequence of the DtxR binding site (**TTAGG**C**AAGGCTA**C**C**T**TT**TTGCCTATG; bold: DtxR binding consensus sequence; underline: mutation C→G; italic: start codon), where DtxR is a repressor of the genes responsible for iron uptake and protection against free radical damage (Wennerhold and Bott, 2006). The mutation on the DtxR binding sequence was assumed to derepress the downstream NCgl1959. To this end, we also found that the transcriptome data show a 5.1-fold upregulation of NCgl1959 in JH41 [Table 2; messenger RNA (mRNA) fold based on transcriptome column]. To verify the hypothesis, the binding affinity between the NCgl1959 regulatory region and DtxR was investigated by gel shift assay (Figure 3A). The binding affinity of the His-tag purified DtxR protein on the DNA fragment carrying the point mutation was only 40% of the affinity of the original sequence, which suggested that the mutation would have caused the derepression of NCgl1959 by reducing affinity to the transcription repressor, DtxR. Further, complementation studies were performed by constitutively expressing NCgl1959, the putative siderophore-binding lipoprotein B, on a plasmid in the PT strain. The PT strain expressing NCgl1959 (PT + pSL360-SBP B) showed more rapid reproduction (75% increase) (Figure 3B), greater iron consumption (Figure 3C), and higher ATP concentrations (Figure 3D) than the parental PT strain. Therefore, the derepression or overexpression of the putative siderophore-binding protein (NCgl1959) enabled *C. glutamicum* to consume more iron and further increase intracellular ATP concentration.

The point mutation found at the -108 region of the NCgl1159 (subunit A of F_0_F_1_-ATP synthase) might be involved in ATP generation (Table 2). The growth profile of the PT host containing the genomic mutation at the -108 region of the NCgl1159 (PT NCgl1159^C–108T^), however, was not different from that of the PT host (Supplementary Figure 3).

**FIGURE 4:**
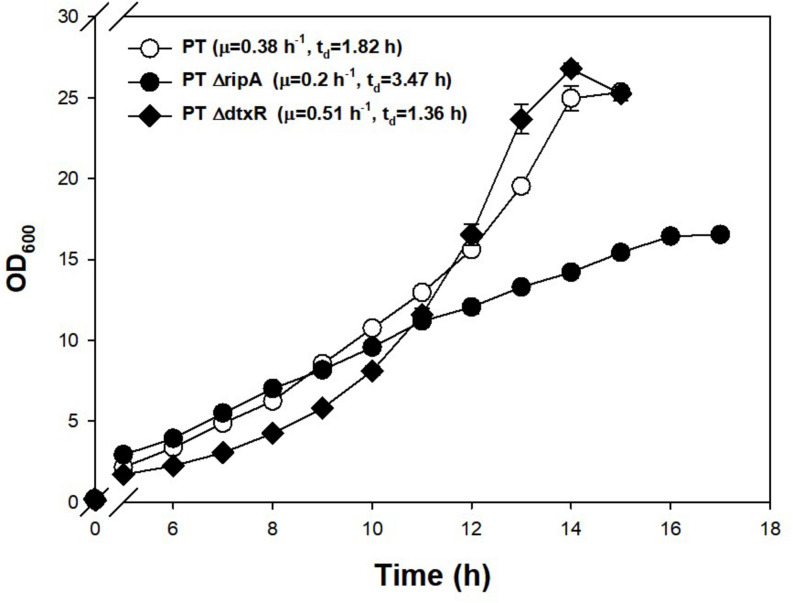
Effect of genomic disruptions of iron-relevant regulator genes (*ripA, dtxR*) on the growth of PT strain. The PT Δ*ripA* and PT Δ*dtxR* strains were incubated at 30°C, 200 rpm in a shaking incubator. A 500-ml baffled flask containing 50 ml of the modified MCGC media was used for efficient oxygen transfer. The exponential growth rate (μ) in batch culture was determined by linear regression of log biomass concentrations over each process time. Each data point is the mean ± SD; *n* = 3 biologically independent samples (PT: white circle; PT Δ*ripA*: black circle; PT Δ*dtxR*: black rhombus).

Investigating further, known regulators related to iron metabolism were found to be disrupted in the PT genome. Along with DtxR, the repressor of the putative siderophore-binding lipoprotein B expression, RipA, which is known to be a gene-enhancing regulator for iron-consuming proteins and is repressed by DtxR (Wennerhold et al., 2005), were chosen as the disruption targets. Part of the coding sequences of DtxR (1.9→1.0 kb) and RipA (1.7→0.9 kb) were removed from the genome of PT strain using a double crossover method. The PT Δ*dtxR* strain showed a 34% faster growth rate and the PT Δ*ripA* (47% slower growth rate) compared with PT strain (Figure 4). The disruption of RipA (leading downregulation of iron-consuming genes) led the cell to double more slowly, and the disruption of DtxR (leading to overexpression of iron-importing genes) resulted in the faster doubling.

### Mutation Effect on Glucose Consumption

**FIGURE 5:**
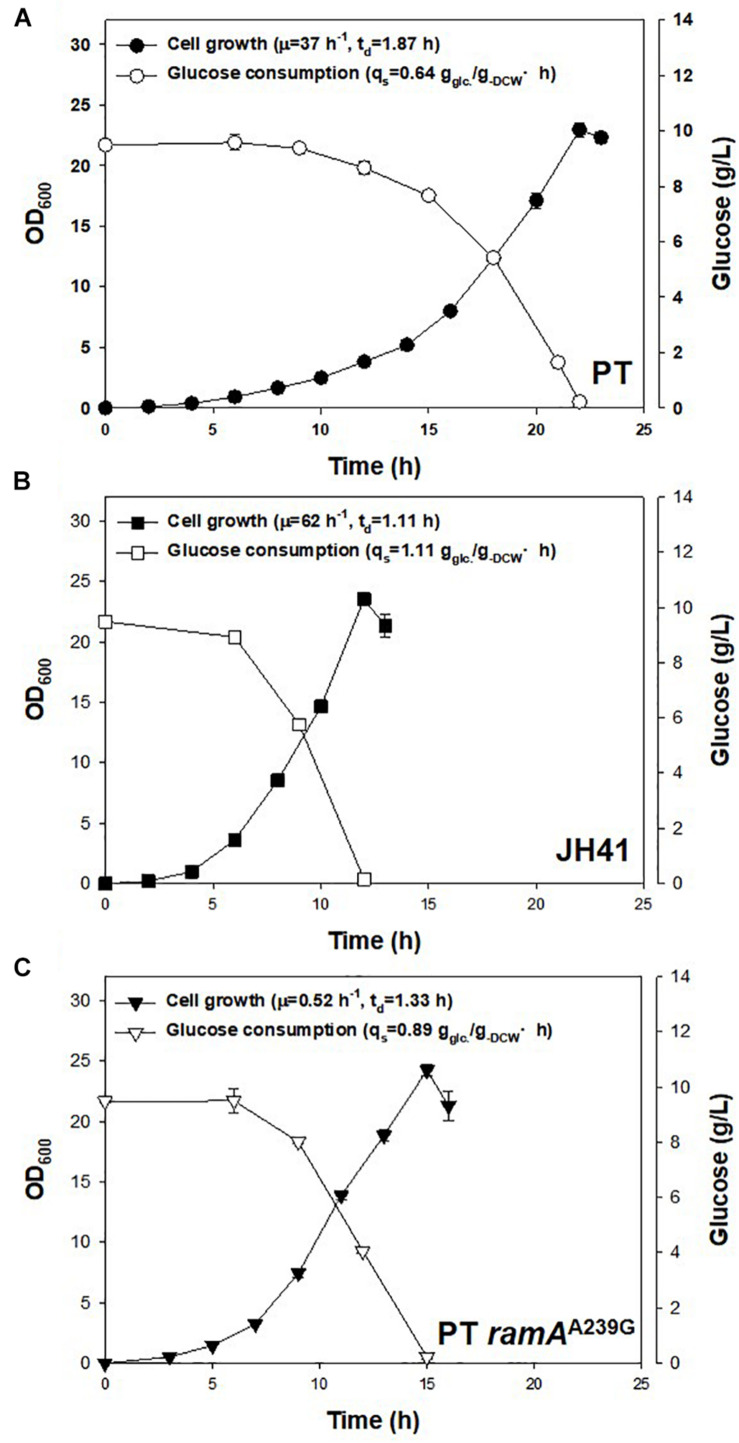
Effects of genomic *ramA*^A239G^ mutation in PT strain on growth rate and glucose consumption. The growth rate and glucose consumption profiles of **(A)** PT, **(B)** JH41, and **(C)** PT *ramA*^A239G^. Batch cultures were performed in a 500-ml baffled flask containing 50 ml of 0.9% glucose modified MCGC medium. Cell densities of *C. glutamicum* PT, JH41, and PT *ramA*^A239G^ were measured every 2 h. Glucose consumption was measured by 3,5-dinitrosalicylic acid (DNS) reduction sugar quantification method. Each data point represents mean ± SD; *n* = 3 biologically independent samples. (biomass: black; glucose concentration: white).

Another notable mutation in JH41 was on the NCgl2472 gene (A239G), encoding the LuxR-type global transcription regulator RamA protein, which controls the expression of genes involved in sugar uptake, glycolysis, acetate, and many other metabolic pathways (Auchter et al., 2011). Wang et al. and Graf et al. reported a *ramA* mutation found in another fast-growing strain of *C. glutamicum*, and the mutation was described as a key mutation because it enabled substrate uptake and increased metabolic flux (Wang et al., 2018; Graf et al., 2019). To verify that the *ramA*^A239G^ mutation in the JH41 strain also contributed to the enhanced cell division, the genomic point mutation was introduced to PT strain by homologous recombination to construct PT *ramA*^A239G^ strain. Indeed, the PT *ramA*^A239G^ strain consumed glucose at 0.89 ± 0.03 g_–glucose_/g_–__DCW_⋅h, whereas PT and JH41 consumed it at 0.64 ± 0.04 and 1.11 ± 0.01 g_–glucose_/g_–__D__CW_⋅h, respectively (Figure 5). Therefore, the *ramA*^A239G^ mutation led the PT strain to increase the rates of glucose consumption by 39% and growth by 40%. The increase in glucose uptake would have provided the increased precursor metabolites and energy to *C. glutamicum* cells.

### Mutation Effect on Ribosome Components

The ITS mutation (condensed 23 point mutations, Table 2) located in the non-coding region of rRNA primary transcript in the genome of JH41 was assumed to influence the maturation of rRNA from its pre-transcript, and this might contribute to the increased number of ribosomes. To confirm this hypothesis, concentrations of the mature 5S, 16S, and 23S rRNAs in the JH41 strain were compared with those in the PT strain by quantitative PCR (Table 3). The fast-growing JH41 strain harbored higher concentrations of mature rRNAs (45% more 16S rRNA, 72% more 23S rRNA, 37% more 5S rRNA) compared to the PT strain. Along with increases in the three rRNAs, our previous paper also reported that most of the mRNAs encoding ribosomal proteins were upregulated (Supplementary Table 2) (Park et al., 2020). These results enabled us to conclude the ITS mutation in rRNA primary transcript supported the fast growth of JH41 by increases in matured rRNAs and ribosomal proteins, and therefore, the increase in ribosome components might be a reverse engineering target to enhance the production of recombinant protein even though the genomic introduction of the multiple mutations in the one ITS region among the 6 rRNA operons would be technically difficult.

**TABLE 3:** Levels of intracellular rRNAs in JH41.

Reference gene	Target gene	Fold difference (2^–ΔΔCT^)^a^
*gapA*	16S rRNA	1.45 ± 0.37
	23S rRNA	1.72 ± 0.64
	5S rRNA	1.37 ± 0.42
*leuA*	16S rRNA	1.47 ± 0.07
	23S rRNA	1.30 ± 0.10
	5S rRNA	1.18 ± 0.15

*^a^(rRNA ratio in JH41)/(rRNA ratio in PT). Values represent the statistical mean ± SD; n = 3 biologically independent samples.*

### Combinatorial Effect of SBP B and RamA Mutations on Recombinant Protein

**FIGURE 6:**
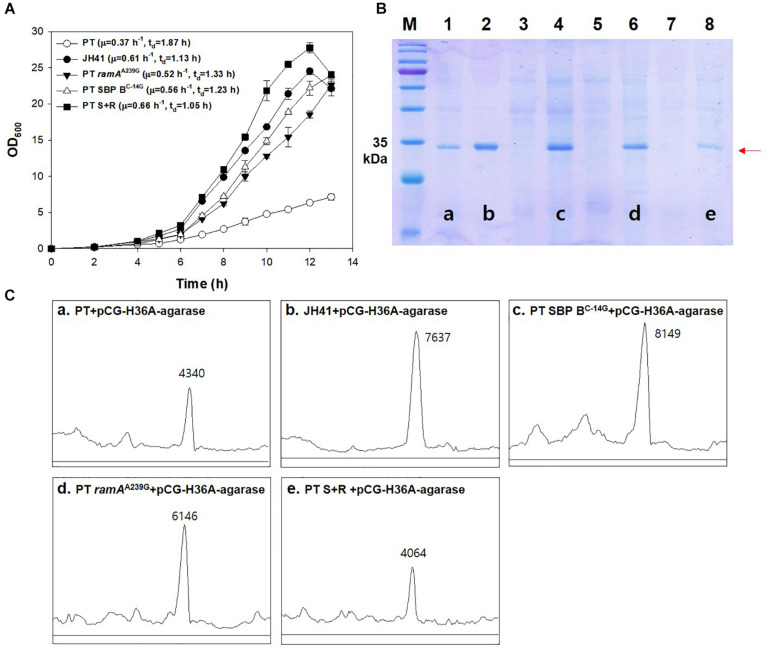
Effects of genomic *ramA*^A239G^ and SBP B^C–14G^ mutation in PT strain on growth rate and the secretory production of recombinant protein. **(A)** Batch culture growth profiles of PT and JH41 strains. The growth rate profiles of PT, JH41, PT *ramA*^A239G^, PT SBP B^C–14G^, and PT S + R. Batch cultures were performed in a 500-ml baffled flask containing 50 ml of 0.9% glucose modified MCGC medium. Cell densities of *C. glutamicum* PT, JH41, PT *ramA*^A239G^, PT SBP B^C–14G^, and PT S + R were measured every 1 h. Each data point represents mean ± SD; *n* = 3 biologically independent samples. (PT: white circle; JH41: black circle; PT *ramA*^A239G^: black triangle; PT SBP B^C–14G^: white triangle; PT S + R: Black square). **(B)** Image of sodium dodecyl sulfate–polyacrylamide gel electrophoresis (SDS-PAGE). Lane 1, PT + pCG-H36A (empty vector); lane 2, JH41 + pCG-H36A-agarase; lane 3, PT SBP B^C–14G^ + pCG-H36A (empty vector); lane 4, PT SBP B^C–14G^ + pCG-H36A-agarase; lane 5, PT *ramA*^A239G^ + pCG-H36A (empty vector); lane 6, PT *ramA*^A239G^ + pCG-H36A-agarase; lane 7, PT S + R + pCG-H36A (empty vector); lane 8, PT S + R + pCG-H36A-agarase. Agarase production was confirmed after 24 h cultivation. Arrow indicates the secreted agarase band (34 kDa). The image is from the representative experiment from three independent experiments. **(C)** Band density estimation of the SDS-PAGE image using ImageJ software. a: lane 1; b: lane 2; c: lane 4; d: lane 6; e: lane 8.

In the above results, the mutations on the SBP B upregulation (SBP B^C–14G^) and the glucose-uptake regulator (*ramA*^A239G^) were the clear genetic factors affecting the growth rate of JH41. To evaluate the combinatorial effect of the two mutations on the growth profile and recombinant protein production, the genome of the PT host was mutated to have either the SBP B^C–14G^ mutation (PT SBP B^C–14G^ host) or the *ramA*^A239G^ mutation (PT *ramA*^A239G^ host) or to have both mutations (PT SBP B^C–14G^ *ramA*^A239G^, in short, PT S + R). The growth rate of the SBP B genome-edited host (PT SBP B^C–14G^) was 0.56 h^–1^ (t_D_ = 1.23 h) and that of the RamA genome-edited host (PT *ramA*^A239G^) was 0.52 h^–1^ (t_D_ = 1.33 h), which were 51 and 41% faster doubling, respectively, than that of PT host (growth rate = 0.37 h^–1^, t_D_ = 1.87 h). The double mutant of *ramA*^A239G^ and SBP B^C–14G^ host (PT S + R) showed the fasted growth rate of 0.66 h^–1^ (t_D_ = 1.05 h), which was 78% higher than that of PT and even faster than that of the evolved JH41 host (0.61 h^–1^, t_D_ = 1.13 h) (Figure 6A).

The secretory protein productions in the PT SBP B^C–14G^ and the PT *ramA*^A239G^ hosts are also shown to be greater than that from the PT host (Figures 6B,C). The SDS-PAGE band intensity of the secretory recombinant proteins from the PT SBP B^C–14G^ and the PT *ramA*^A239G^ hosts were 8,149 and 6,146 AU, respectively, where those from PT host was 4,130 AU. The fastest growing PT S + R hosts, however, secreted the recombinant protein at only 4,064 AU, which is even lower than that of the PT host. Therefore, the mutations in *ramA*^A239G^ and SBP B^C–14G^, respectively, resulted in the increased growth rates and protein production. Meanwhile, the combination of the two genes showed a remarkable increase in the growth rate but was found to have a negative effect on protein production. The iron metabolism regulator-deleted strains (PT Δ*dtxR* and PT Δ*ripA*) also decreased the recombinant agarase production by 34 and 37%, respectively, than that from PT host (Supplementary Figure 4).

## Discussion

The evolved *C. glutamicum* JH41 strain with increased growth rate was able to produce 2.7-fold greater secretory recombinant protein than PT host (Figure 1). The confirmed genetic factors that contributed to the increased growth rates and became the reverse engineering targets to enhance production of recombinant proteins were (1) SBP B^C–14G^ that enhanced iron transport leading the increase of intracellular energy and (2) *ramA*^A239G^ that enhanced glucose uptake leading to the increase in energy and metabolite pool. High levels of intracellular ATP have been known to improve target products by improving substrate uptake, cell growth, biosynthesis, and resistance to toxic compounds (Hara and Kondo, 2015). The authors also reported that the productions of recombinant proteins (green fluorescence protein and alkaline protease) were increased in the highly energized *E. coli* host by the overexpression of phosphoenolpyruvate carboxykinase (Pck) (Kim et al., 2012). A high level of intracellular energy can provide favorable conditions for energy-consuming reactions such as charging amino acids to transfer RNA (tRNA), translational elongation, folding, and secretion. In addition, a high intracellular energy state can upregulate the genes involved in the biosynthetic pathways for amino acid supply (Kwon et al., 2008).

The point substitution mutation in the regulatory region of NCgl1959 in JH41 strain, resulted in the increase in iron consumption by derepressing the cognate genes (Figure 3). Although the function of NCgl1959 protein in *C. glutamicum* has not yet been clearly identified, the NCgl1959 protein was estimated to have a similar 3D structure with the known siderophore-binding lipoproteins (*E. coli* FhuD and *Bacillus cereus* YfiY) (Clarke et al., 2000; Zawadzka et al., 2009) (Supplementary Figure 5). One might speculate that NCgl1959 would play the role of iron complex uptake. The imported iron could be used for the assembly of electron transfer chain components (i.e., iron–sulfur reaction center for NADH dehydrogenase; heme–iron for cytochrome *c* oxidase and cytochromes) and redox-containing proteins (i.e., heme-dependent catalase and peroxidase) (Park et al., 2020), and therefore, it is considered that the enhanced iron consumption in JH41 strain would contribute to generating more energy to the respiratory chain, along with a simultaneous increase in defense capability against oxidative stress.

In particular, RamA has been shown to act as a positive regulator of the TCA cycle genes, *sdhCAB* (succinate dehydrogenase operon) and *acn* (aconitase) (Bussmann et al., 2009; Teramoto et al., 2011). The point mutation *ramA*^A239G^ (corresponding to RamA^Y80C^) of JH41 strain was located at the GAF-2 domain (amino acid positions 8 and 146), the same domain of the reports from Graf et al. and Wang et al. (Wang et al., 2018; Graf et al., 2019), and the GAF domain has been found in the cyclic nucleotide phosphodiesterase superfamily in other species (Fawcett et al., 2000; Schultz, 2009). Therefore, we considered that the *ramA*^A239G^ would have enabled the JH41 strain to increase glucose consumption by altering the cyclic nucleotide-mediated signals, thereby resulting in increased substrate uptake. The increase in carbon source in the JH41 host would have been favorable cellular conditions for the recombinant protein production by providing enhanced pools of precursor metabolites, along with more energy.

We do not understand why the combination of the two mutations of *ramA*^A239G^ and SBP B^C–14G^ showed a faster growth rate exceeding JH41 and less production of recombinant protein (Figure 6). The H36 promoter for the recombinant protein production was one of the most powerful synthetic promoter known in *C. glutamicum* (Yim et al., 2013), and it was able to promote the secretory production of agarase from the PT *ramA*^A239G^ and the PT SBP B^C–14G^ along with the JH41 hosts. There might be unknown factor responsible for this mismatch between fast growth and less protein production (Nordholt et al., 2017). Further basic studies on cellular physiology are required to understand this unexpected phenomenon.

The condensed 23 point mutations occurred in the ITS region of the first rRNA operon among the six rRNA operons of *C. glutamicum* JH41. The multimutated first ITS sequence was similar to the sequence of the ITS in the fifth rRNA operon. A tRNA gene is located between 16S rRNA and 23S rRNA in *E. coli* rRNA pretranscript, while *C. glutamicum* does not contain tRNA in this sequence region. Although the processing mechanism of the rRNA pretranscript in *C. glutamicum* remains unknown, the ITS mutation in JH41 might have facilitated RNase processing, as in the case of *E. coli*. Therefore, the processing of the polycistronic primary transcript might have been increased to form mature ribosomes by the ITS mutation, having observed the increases in rRNAs and ribosomal proteins. The increase in mature ribosome components would contribute to the enhancement of recombinant protein productions, as observed in JH41.

Additional genetic factors enhancing growth and recombinant protein production remain veiled. It is worth mentioning another complementation on the genomic mutation on the coding region of NCgl2298 (*osrR*, G757A = D253N), an HTH-type transcription regulator known to upregulate the tolerance genes against oxidative stress and heat stress (Hong et al., 2016). The mutation on the *osrR* was expected to release the stress tension that was provoked by the enhanced iron and carbon uptakes. The growth of the PT NCgl2298^G757A^ was, however, no better than that of the PT strain (Supplementary Figure 6A). The combination of mutations (i.e., the mutations on *ramA* + *osrR*) would be required for further understanding.

Besides, a mutation in the -109 region of NCgl1159 (F_0_F_1_-ATP synthase subunit A), which was expected to affect cell growth by altering ATP generation, did not increase the growth rate (Supplementary Figure 3). The transcription levels of ATP synthase components in JH41 were only half those in our previous paper (Park et al., 2020), so the half-expressed ATP synthase might have been somehow balanced for the enhanced energy generation under the increased iron and carbon uptake conditions. Another possible mutation to consider is NCgl2331, a penicillin-binding protein involved in cell defense. Substituting A for the 610th G of the NCgl2331 gene in the PT strain resulted in marginal increase (14%) in growth rate (Supplementary Figure 6B). Penicillin-binding proteins are membrane-associated macromolecules and are expected to play a key role in cell biosynthesis and cell division (Macheboeuf et al., 2006).

In this respect, our discovery offers essential cues for the construction of an efficient cell factory for the production of recombinant proteins and expands the biological knowledge base. Studies on the reverse engineering of the other mutations including the ITS condensed mutations might lead us to further improvement on *C. glutamicum* host.

## Data Availability Statement

The JH41 genome was resequenced and deposited in NCBI accession number: PRJNA554987.

## Author Contributions

ML, JP, and KP performed the experiments. ML and PK designed the experiments. JK and PK raised grant and supervised. ML and PK wrote the manuscript. All authors contributed to the article and approved the submitted version.

## Conflict of Interest

The authors declare that the research was conducted in the absence of any commercial or financial relationships that could be construed as a potential conflict of interest.
