# Supplementary material for: Reverse Engineering Targets for Recombinant Protein Production in Corynebacterium glutamicum Inspired by a Fast-Growing Evolved Descendant
Source: Front Bioeng Biotechnol. 2020 Dec 9;8:588070. doi: 10.3389/fbioe.2020.588070 (PMC7755716; doi:10.3389/fbioe.2020.588070)
Supplement: Supplementary file 1 [file Table_1.docx]

***Supplementary Material***

**Table S1. Oligonucleotides used in this study.**

| Oligonucleotide | Sequence (5’-3’) | Restriction site | Target gene |
| --- | --- | --- | --- |
| P1 | GGA TCC AAA GGA GGA CAA ATG AGC AAA GGA GAA | *BamHI* | GFP (pCG-H36A(-SS)-GFP) |
| P2 | ACT AGT TTA TTT GTA GAG CTC | *SpeI* |  |
| P3 | GGA TCC ATG CCA CAG TTA AGC A | *BamHI* | *porD* signal peptide (pCG-H36A-*porD*) |
| P4 | GGA TCC TTG GCG TTC TTC AGC G | *BamHI* |  |
| P5 | CAT ATG GTG AAG GAT CTG GT | *NdeI* | DtxR (pET24a-DtxR) |
| P6 | AAA AGG TTG AGG GCC TCG AG | *XhoI* |  |
| P7 | TCG TCA GCA TTT GAA | *-* | NCgl1959 regulatory region |
| P8 | GTT GAG GGC GAC GAT | *-* |  |
| P9 | GTC GAC AGG AGG AAA ATG CAT ATG GAG ATT | *SalI* | SBP (pSL360-SBP B) |
| P10 | GCG GCC GCC TAG ACG GTG ACG TTG TCT | *NotI* |  |
| P11 | AAG CTT CGC GGG CGG CAG GTT | *HindIII* | Homologous arms flanking the 1,000 bp insertion fragment inside NCgl0774  (pK19mobsacB-NCgl0774::35-bp) |
| P12 | TCT AGA TTT ATC GTT AAA GCC | *XbaI* |  |
| P13 | TCT AGA GGG TAT TTT CGG TAG CGT AT | *XbaI* | Homologous arms flanking the 800 bp deletion fragment inside *ripA*  (pK19mobsacB-Δ*ripA*) |
| P14 | TTT TCA CAC CTT TAC TAC CTA TCT CAT CCT CAC TAC AAG C | *-* |  |
| P15 | GCT TGT AGT GAG GAT GAG ATA GGT AGT AAA GGT GTG AAA A | *-* |  |
| P16 | GTC GAC AGA TCC GTA CTT CTT GCC GA | *SalI* |  |

| Oligonucleotide | Sequence (5’-3’) | Restriction site | Target gene |
| --- | --- | --- | --- |
| P17 | TCT AGA GAG CGT GAA GTC ATG AGT AA | *XbaI* | Homologous arms flanking the 900 bp deletion fragment inside *dtxR* (pK19mobsacB-Δ*dtxR*) |
| P18 | AGA AGC TTC ATC GTG TGT TAG AGG CTG CCT TCC TTG TTT T | *-* |  |
| P19 | AAA ACA AGG AAG GCA GCC TCT AAC ACA CGA TGA AGC TTC T | *-* |  |
| P20 | GTC GAC TTT CAC CGA TGT TTC CGT AT | *Sal I* |  |
| P21 | AAG CTT TCT AAA ACC GGC GTG | *HindIII* | Homologous arms flanking the 1,000 bp substitution fragment inside *ramA*  (pK19mobsacB-*ramA*^A239G^) |
| P22 | TCT AGA CGA TGC CCA TCT CTT | *XbaI* |  |
| P23 | AAG CTT CCA ACG TTC CAG GAA T | *HindIII* | Homologous arms flanking the 1,000 bp substitution fragment 108 upstream from NCg1959 coding region (pK19mobsacB-NCgl1159^C-14G^) |
| P24 | TCT AGA GTG AGG AGG TCT TGC | *XbaI* |  |
| P25 | CTG CAG CTT GAA ACA GGG GAG | *PstI* | Homologous arms flanking the 1,000 bp substitution fragment 108 upstream from NCg1159 coding region (pK19mobsacB-NCgl1159^C-108T^) |
| P26 | GGA TCC AAT ACG TGA GTT GGA | *BamHI* |  |
| P27 | AAGCTTCCGGTGCGCTTAACCTCT | *HindIII* | Homologous arms flanking the 1,000 bp substitution fragment inside NCgl2331  (pK19mobsacB-NCgl2331^G610A^) |
| P28 | TCT AGA TGC CTC CTC CAA TGA GGA | *XbaI* |  |

**Table S1. Continued.**

| Oligonucleotide | Sequence (5’-3’) | Restriction site | Target gene |
| --- | --- | --- | --- |
| P29 | AAG CTT TGC GCC CAG AGG CTG | *HindIII* | Homologous arms flanking the 1,000 bp substitution fragment inside NCgl2298  (pK19mobsacB-NCgl2298^G757A^) |
| P30 | TCT AGA GAG TGG TTC CGA TTC | *XbaI* |  |
| P31 | TGG TTC ACA CCG TAA ACG AA |  | qPCR primer-*gapA* |
| P32 | GCT AAG GCT CAC ATC GAA GC |  |  |
| P33 | TTT CGG TTT GAG CAT TTT CC |  | qPCR primer- *leuA* |
| P34 | GGT CAA CTC CAA GGC AAT GT |  |  |
| P35 | TCG TAG GTG GTT TGT CGC |  | qPCR primer- 16SrRNA |
| P36 | CAC TCA AGT TAT GCC CGT AT |  |  |
| P37 | CCT GCC GTA GAA GGT GAG AG |  | qPCR primer- 23SrRNA |
| P38 | TTC ACA GCA GAT TCC ACG AG |  |  |
| P39 | GTC GGT GGT GAT AGT AGC |  | qPCR primer- 5SrRNA |
| P40 | GGT CGG CGG TAA CCT ACT |  |  |

**Table S1. Continued.**

**Figure S1. Effect of host on the cytoplasmic (A) and secretory (B) production of GFP.**

**(A) Cytoplasmic production of GFP**

Cells were incubated at 30℃ and 200 rpm in a shaking incubator. A 500-mL baffled flask containing 50 mL of the modified MCGC media was used for efficient oxygen transfer. After collecting the cell broth every 12 hours, supernatant was removed and resuspended in Tris-HCl (pH 7.0) to measure fluorescence. Each data point is the mean ± SD; n=3 biologically independent samples.

**(B) Secretory production of GFP**

Cells were incubated at 30℃ and 200 rpm in a shaking incubator. A 500-mL baffled flask containing 50 mL of the modified MCGC media was used for efficient oxygen transfer. After collecting the cell broth every 12 hours, the cells were discarded by centrifuge and the supernatant was taken to measure the fluorescence. Each data point is the mean ± SD; n=3 biologically independent samples.

**(Method)** PT hosts harboring the pCG-H36(-SS)-GFP (cytoplasmic GFP expression) and the pCG-H36-*porD*-GFP (secretory GFP expression) were cultured in MCGC media for 48 h. Cells were harvested every 12 hours by centrifugation (7,000 rpm, 10 min). The cells were resuspended in a Tris-HCl (pH7.0) buffer to measure the fluorescence of the cytosolic protein. The secreted protein was confirmed by the fluorescence measurement of the supernatant obtained after harvesting the cells. Samples (200 μl) were dispensed into a 96-well black plate and subjected to a fluorescence spectrophotometer (Synergy MX, BioTek, VT, USA) (Ex. 491 nm, Em. 511 nm)

**Figure S2. Effect of putative siderophore-binding lipoprotein A disruption (PT NCgl0774::35-bp strain) on the reproduction rate.**

Cells were incubated at 30℃ and 200 rpm in a shaking incubator. A 500-mL baffled flask containing 50 mL of the modified MCGC media was used for efficient oxygen transfer. The exponential growth rate (μ) in batch culture was determined by linear regression of log biomass concentrations over each process time. Each data point is the mean ± SD; n=3 biologically independent samples.

**Figure S3. Effect of genomic NCgl1159 mutation (PT NCgl1159^C-108T^strain) on growth rate in PT strain.**

Cells were incubated at 30℃ and 200 rpm in a shaking incubator. A 500-mL baffled flask containing 50 mL of the modified MCGC media was used for efficient oxygen transfer. The exponential growth rate (μ) in batch culture was determined by linear regression of log biomass concentrations over each process time. Each data point is the mean ± SD; n=3 biologically independent samples.

**Table S2. Selected mRNA levels of ribosomal proteins in JH41 strain**

| **Gene name** | **Locus tag** | **Function** | **^1)^mRNA fold** |
| --- | --- | --- | --- |
| *rpmH* | NCgl2993 | Large subunit ribosomal protein L34 | 2.46 |
| *rpmA* | NCgl2279 | Large subunit ribosomal protein L27 | 1.80 |
| *rplU* | NCgl2280 | Large subunit ribosomal protein L21 | 1.74 |
| *rpmB* | NCgl0834 | Large subunit ribosomal protein L28 | 1.73 |
| *rplA* | NCgl0460 | Large subunit ribosomal protein L1 | 1.66 |
| *rpmI* | NCgl1325 | Large subunit ribosomal protein L35 | 1.65 |
| *rplJ* | NCgl0468 | Large subunit ribosomal protein L10 | 1.64 |
| *rplM* | NCgl0556 | Large subunit ribosomal protein L13 | 1.63 |
| *rpsE* | NCgl0518 | Small subunit ribosomal protein S5 | 1.61 |
| *rplR* | NCgl0517 | Large subunit ribosomal protein L18 | 1.58 |
| *rpsI* | NCgl0557 | Small subunit ribosomal protein S9 | 1.57 |
| *rpsH* | NCgl0515 | Small subunit ribosomal protein S8 | 1.56 |
| *rplY* | NCgl0902 | Large subunit ribosomal protein L25 | 1.56 |
| *rpsR* | NCgl0831 | Small subunit ribosomal protein S18 | 1.54 |
| *rplK* | NCgl0459 | Large subunit ribosomal protein L11 | 1.54 |
| *rplE* | NCgl0501 | Large subunit ribosomal protein L5 | 1.50 |
| *rplO* | NCgl0520 | Large subunit ribosomal protein L15 | 1.50 |
| *rpsQ* | NCgl0496 | Small subunit ribosomal protein S17 | 1.49 |
| *rplL* | NCgl0469 | Large subunit ribosomal protein L7/L12 | 1.48 |
| *rplS* | NCgl1960 | Large subunit ribosomal protein L19 | 1.48 |
| *rpmF* | NCgl0838 | Large subunit ribosomal protein L32 | 1.47 |
| *rpsO* | NCgl1901 | Small subunit ribosomal protein S15 | 1.47 |
| *rpsN* | NCgl0832 | Small subunit ribosomal protein S14 | 1.46 |
| *rplF* | NCgl0516 | Large subunit ribosomal protein L6 | 1.45 |
| *rpmJ* | NCgl2446a | Large subunit ribosomal protein L36 | 1.43 |
| *rplX* | NCgl0500 | Large subunit ribosomal protein L24 | 1.42 |
| *rpsA* | NCgl1304 | Small subunit ribosomal protein S1 | 1.42 |
| *rplN* | NCgl0499 | Large subunit ribosomal protein L14 | 1.41 |
| *rpsG* | NCgl0477 | Small subunit ribosomal protein S7 | 1.40 |
| *rpsB* | NCgl1950 | Small subunit ribosomal protein S2 | 1.38 |
| *rpmE* | NCgl0837 | Large subunit ribosomal protein L31 | 1.37 |
| *rpsL* | NCgl0476 | Small subunit ribosomal protein S12 | 1.35 |
| *rpsT* | NCgl2261 | Small subunit ribosomal protein S20 | 1.35 |
| *rpsP* | NCgl1976 | Small subunit ribosomal protein S16 | 1.30 |
| *rplQ* | NCgl0541 | Large subunit ribosomal protein L17 | 1.30 |
| *rpmG* | NCgl0833 | Large subunit ribosomal protein L33 | 1.30 |

**Table S2. Continued.**

| **Gene name** | **Locus tag** | **Function** | **^1)^mRNA fold** |
| --- | --- | --- | --- |
| *rpmG* | NCgl0833 | Large subunit ribosomal protein L33 | 1.30 |
| *rpmC* | NCgl0495 | Large subunit ribosomal protein L29 | 1.27 |
| *rpmD* | NCgl0519 | Large subunit ribosomal protein L30 | 1.24 |
| *rpsD* | NCgl0539 | Small subunit ribosomal protein S4 | 1.20 |
| *rplT* | NCgl1326 | Large subunit ribosomal protein L20 | 1.16 |
| *rplP* | NCgl0494 | Large subunit ribosomal protein L16 | 1.11 |
| *rplI* | NCgl2879 | Large subunit ribosomal protein L9 | 1.10 |
| *rpsM* | NCgl0537 | Small subunit ribosomal protein S13 | 1.04 |
| *rpsF* | NCgl2881 | Small subunit ribosomal protein S6 | 1.04 |
| *rpsK* | NCgl0538 | Small subunit ribosomal protein S11 | 1.00 |
| *rpsC* | NCgl0493 | Small subunit ribosomal protein S3 | 0.99 |
| *rplV* | NCgl0492 | Large subunit ribosomal protein L22 | 0.92 |
| *rpsS* | NCgl0491 | Small subunit ribosomal protein S19 | 0.87 |
| *rplW* | NCgl0489 | Large subunit ribosomal protein L23 | 0.85 |
| *rplB* | NCgl0490 | Large subunit ribosomal protein L2 | 0.83 |
| *rplD* | NCgl0488 | Large subunit ribosomal protein L4 | 0.81 |
| *rpsJ* | NCgl0486 | Small subunit ribosomal protein S10 | 0.80 |
| *rplC* | NCgl0487 | Large subunit ribosomal protein L3 | 0.72 |

^1)^ JH41/PT mRNA ratio

The data were adopted from the transcriptome data ([Park et al., 2020](#_ENREF_1)), which are available at NCBI with access code (PRJNA556334).

**Figure S4. Effect of Δ*dtxR* and Δ*ripA* on the secretory production of recombinant protein**

**(A) Image of SDS-PAGE.**

Lane 1, PT+pCG-H36A-agarase at 24 h; Lane 2, JH41+pCG-H36A-agarase at 24 h; Lane 3, PT Δ*dtxR*+pCG-H36A (empty vector) at 24 h; Lane 4, PT Δ*dtxR*+pCG-H36A-agarase at 24 h; Lane 5, PT Δ*ripA*+pCG-H36A (empty vector) at 24 h; Lane 6, PT Δ*ripA*+pCG-H36A-agarase at 24 hours. Arrow indicates the secreted agarase band (34 kDa). The image is from the representative experiment from three independent experiments.

**(B) Band density estimation of the SDS-PAGE image using ImageJ software.**

a: lane 1; b: lane 2; c: lane 4; d: lane 6;

**Figure S5. 3D SWISS-MODEL of the NCgl1959 compared with reported siderophore-binding proteins**

1. Estimated 3D structure of NCgl1959 from *C. glutamicum*
2. PDB structure of a siderophore-binding protein from *E. coli*
3. PDB structure of a siderophore-binding protein from *B. cereus*

**Figure S6. Effect of the genomic mutations at NCgl2298^G757A^ (A), and NCgl2331^G610A^ (B) on the growth of *C. glutamicum*.**

Cells were incubated at 30℃ and 200 rpm in a shaking incubator. A 500-mL baffled flask containing 50 mL of the modified MCGC media was used for efficient oxygen transfer. The exponential growth rate (μ) in batch culture was determined by linear regression of log biomass concentrations over each process time. Each data point is the mean ± SD; n=3 biologically independent samples.
